# Two *N*,*N*′-bis­(pyridin-4-yl)pyridine-2,6-dicarboxamide coordination compounds

**DOI:** 10.1107/S2056989018007351

**Published:** 2018-07-06

**Authors:** Yue-Xin Guo, Hong-Cui Ma, Ren Bo, Ning Zhao, Li-Gang Zhao, Jin-Peng Li, Hong-Wei Hou

**Affiliations:** a School of Pharmacy, North China University of Science and Technology, Tangshan, 063210, Hebei, People’s Republic of China; b College of Chemistry and Molecular Engineering, Zhengzhou University, Zhengzhou, 450001, Henan, People’s Republic of China

**Keywords:** crystal structure, coordination compound, heterocyclic nitro­gen ligand, Mn^II^ complex, Cd^II^ complex

## Abstract

The molecular structures of tetra­aqua­[*N*,*N*′-bis­(pyridin-4-yl)pyridine-2,6-dicar­boxamide]­sulfatomanganese(II) dihydrate, [Mn(SO_4_)(C_17_H_13_N_5_O_2_)(H_2_O)_4_]·2H_2_O or [Mn(H_2_ *L* ^1^)(SO_4_)(H_2_O)_4_]·2H_2_O, (I), and tetra­aqua­bis[*N*,*N*′-bis­(pyridin-4-yl)pyridine-2,6-dicar­boxamide]cadmium(II) sulfate tetra­hydrate, [Cd(C_17_H_13_N_5_O_2_)_2_(H_2_O)_4_]SO_4_·4H_2_O or [Cd(H_2_O)_4_(H_2_ *L* ^1^)_2_]·SO_4_·4H_2_O, (II), both contain a central metal atom in a distorted octa­hedral geometry coordinated equatorially by four oxygen atoms from water mol­ecules. In (I), the axial positions are occupied by a nitro­gen atom from H_2_ *L* ^1^ and an oxygen atom from the sulfate anion, whereas in (II), the axial positions contain two nitro­gen atoms from two different H_2_ *L* ^1^ ligands and the sulfate anion acts as the charge-balancing ion. π–π stacking between pyridine rings and a network of hydrogen bonds involving the water molecules and the sulfate anions play a crucial role in the mol­ecular self-assembly of the two structures.

## Abstract 2

The title compounds both contain a central metal atom in a distorted octa­hedral geometry coordinated equatorially by four oxygen atoms from water mol­ecules. In the Mn^II^ complex, the axial positions are occupied by a nitro­gen atom from the ligand and an oxygen atom from the sulfate anion, whereas in the Cd^II^ complex they contain two nitro­gen atoms from two different ligands and the sulfate anion only serves as the charge-balancing ion. π–π stacking between pyridine rings plays a crucial role in the mol­ecular self-assembly of the two structures.

## Chemical context   

In recent years, the design of metal–organic complexes constructed from heterocyclic nitro­gen-derivative ligands has witnessed an upsurge in inter­est due to their fascinating structures and potential applications in luminescence, catal­ysis, gas storage and separation (Perry *et al.*, 2004 ▸). The heterocyclic nitro­gen-derivative ligands have σ-electron-donating ability and can form strong *M*—N covalent bonds with transition-metal ions. This bonding feature, when combined with the flexibility and length of mol­ecular backbone within these ligands, can lead to the construction of porous coordination compounds (Gao *et al.*, 2003 ▸; Hagrman *et al.*, 1999 ▸; Li *et al.*, 2017 ▸). A variety of heterocyclic nitro­gen-derivative complexes with inter­esting properties and topologies have been synthesized using ligands such as 4,4′-bi­pyridine (Fujita *et al.*, 1994 ▸), an asymmetric triazole di­carboxyl­ate ligand (Hao *et al.*, 2018 ▸), 5-(pyridine-3-yl)pyrazole-3-carb­oxy­lic acid (Cheng *et al.*, 2016 ▸), 2-[4-(1*H*-imidazole-1-ylmeth­yl)-1*H*-1,2,3-triazol-1-yl] acetic acid (Yu *et al.*, 2016 ▸) and 2,2′-dihy­droxy-[1,1′]binaphthalenyl-3,3′-di­carb­oxy­l­ate (Zheng *et al.*, 2004 ▸). Heterocyclic ligands containing aromatic systems continue to attract our inter­est because they can form various π–π stacking inter­actions between pyridine rings and direct the crystal packing and mol­ecular assembly (Tomura & Yamashita, 2001 ▸; Li *et al.*, 2012 ▸). Here we report the synthesis and crystal structures of two new mononuclear complexes, [Mn(H_2_ *L* ^1^)(SO_4_)(H_2_O)_4_]·2H_2_O, (I), and [Cd(H_2_ *L* ^1^)_2_(H_2_O)_4_]SO_4_·4H_2_O, (II), both of which contain *N*,*N*′-bis­(pyridin-4-yl)pyridine-2,6-dicarboxamide (H_2_ *L* ^1^) as the heterocyclic nitro­gen ligand.
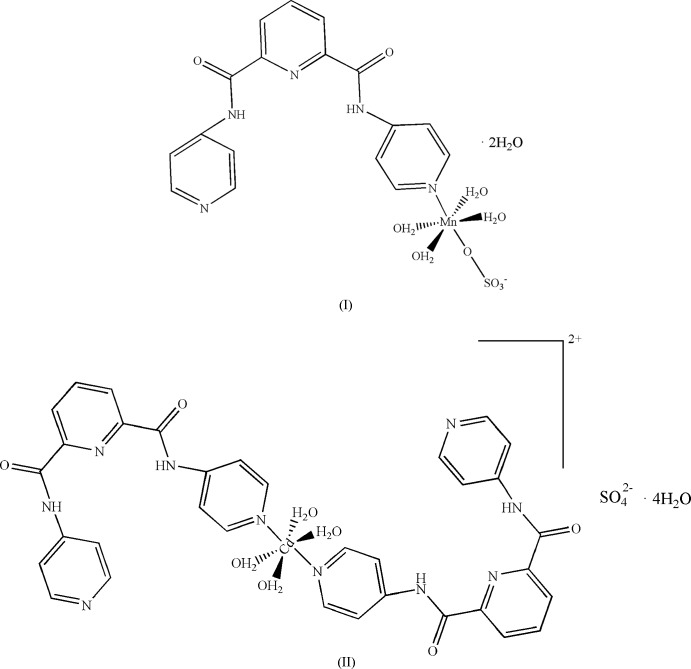


In complexes (I) and (II), the heterocyclic nitro­gen ligand (H_2_ *L* ^1^) acts as a monodentate ligand. The results indicate that the rational design and selection of ligands with heterocyclic nitro­gen systems is an effective synthetic strategy to construct complexes *via* self-assembly. The asymmetry of the [*N*,*N*′-bis­(pyridin-4-yl)pyridine-2,6-dicarboxamide ligand has resulted in some novel structures (Li *et al.*, 2012 ▸). Further study is ongoing.

## Structural commentary   

**Figure 1:**
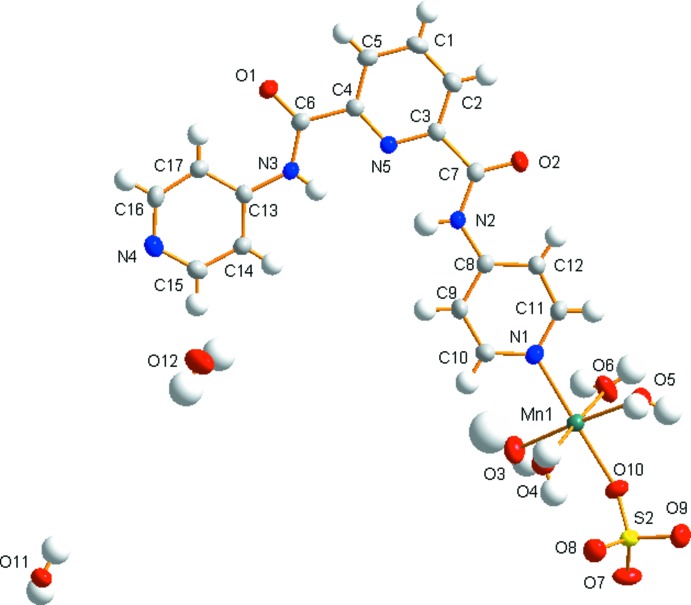
The mol­ecular structure of the title complex (I) with displacement ellipsoids shown at the 50% probability level.

The mononuclear complex (I) crystallizes in the triclinic space group *P* 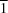. As shown in Fig. 1 ▸, the hexa­coordinated Mn^II^ ion exhibits an octa­hedral geometry, arising from coordination to five water and one sulfate oxygen atoms (O3, O4, O5, O6, O10) and to one nitro­gen (N1) atom of a pyridine group of the H_2_ *L* ^1^ ligand (Table 1 ▸). The Mn—O bond distances involving the water mol­ecules coordinated by manganese(II) in equatorial positions lie in the range 2.1502 (17)–2.2333 (17) Å whilst the Mn—N1 and Mn—O10 distances in the axial positions are 2.2208 (17) and 2.1492 (15) Å, respectively. The bond angles around the Mn^II^ ion vary from 85.79 (7) to 177.74 (6)°. Intra­molecular O4—H4⋯O8, N2—H20⋯N5 and N3—H24⋯N5 hydrogen bonds are also present (Table 3 ▸).

**Figure 2:**
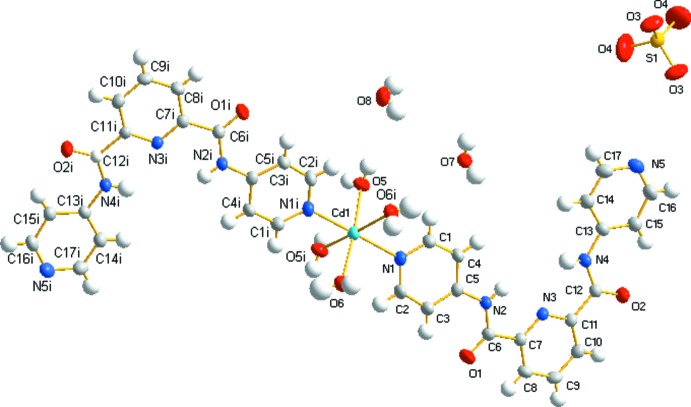
The mol­ecular structure of the title complex (II) with displacement ellipsoids shown at the 50% probability level. Symmetry code: (i) −*x*, *y*, −*z* + 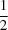.

When MnSO_4_·H_2_O is replaced by CdSO_4_·8/3H_2_O, complex (II) is obtained, which crystallizes in the monoclinic space group *C*2/*c*. As illustrated in Fig. 2 ▸, Cd^II^ also shows an octahedral environment coordinating four oxygen atoms from four water molecules and two axial nitrogen atoms from two symmetry-related H_2_ *L* ^1^ ligands (Table 2 ▸). In contrast to complex (I), the sulfate group does not coordinate to the cadmium(II) atom, but balances the compound charge as a free anion. The Cd—O bond lengths lie in the range 2.334 (2)– 2.371 (3) Å, the Cd—N bond length is 2.275 (3) Å, and the bond angles around the Cd^II^ cation lie in the range 82.63 (14) to 175.09 (10)°. Intra­molecular N2—H2*B*⋯N3 and N4—H4*B*⋯N3 hydrogen bonds are also present (Table 4 ▸).

In complexes (I) and (II), the heterocyclic nitro­gen ligand (H_2_ *L* ^1^) acts as a monodentate ligand. The results indicate that the rational design and selection of ligands with heterocyclic nitro­gen systems is an effective synthetic strategy to construct complexes *via* self-assembly. The asymmetry of the [*N*,*N*′-bis­(pyridin-4-yl)pyridine-2,6-dicarboxamide ligand has resulted in some novel structures. Further study is ongoing.

## Supra­molecular features   

**Figure 3:**
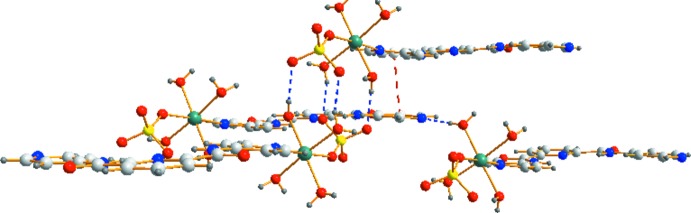
The weak inter­actions between mol­ecules of complex (I). π–π inter­actions are shown as red dashed lines, hydrogen-bonding inter­actions as blue dashed lines.

**Figure 4:**
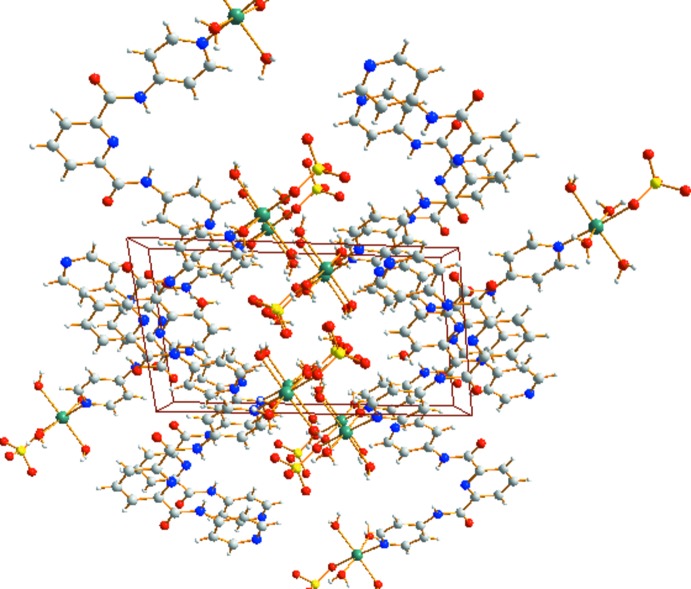
The mol­ecular packing of (I) viewed along the *a* axis.

In (I), inter­molecular π–π inter­actions between the pyridine rings of the H_2_ *L* ^1^ ligands play a crucial role in mol­ecular self-assembly, with centroid-to-centroid separations of 3.5808 (13) and 3.6269 (14) Å. In addition, a number of O—H⋯N and O—H⋯O hydrogen-bonding inter­actions (Table 3 ▸ and Fig. 3 ▸) connect separate mononuclear structures to produce a three-dimensional supra­molecular framework (Fig. 4 ▸).

**Figure 5:**
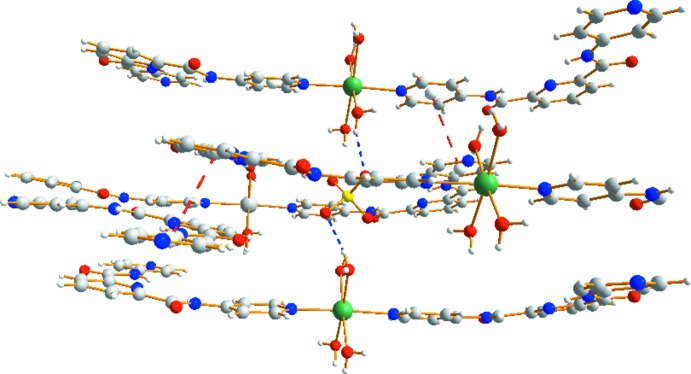
The weak inter­actions between mol­ecules of complex (II) with hydrogen-bonding inter­actions and π–π interactions shown as blue and red dashed lines, respectively.

Complex (II) also extends into a three-dimensional supra­molecular network (Figs. 5 ▸ and 6 ▸) *via* O—H⋯N and O—H⋯O hydrogen-bonding inter­actions (Table 4 ▸). In (II), the centroid–centroid separations of the pyridine rings are 3.634 (2) and 3.768 (2) Å and indicating that inter­molecular π–π stacking inter­actions of the H_2_ *L* ^1^ ligand are important in molecular self-assembly.

## Database survey   

A search of the Cambridge Crystallographic Database (CSD, version 5.39, update May 2018; Groom *et al.*, 2016 ▸) reveals eight structures with the H_2_ *L* ^1^ skeleton. These include the methyl pyridinium compound of H_2_ *L* ^1^ (Dorazco-González *et al.*, 2010 ▸), two Ru^+^ compounds (Park *et al.*, 2006 ▸; Mishra *et al.*, 2012 ▸), two Pd^2+^ compounds (Qin *et al.*, 2002 ▸, 2003 ▸) and two Co^2+^ compounds (Singh *et al.*, 2010 ▸, 2011 ▸). There is only one Mn^2+^ coordination compound (Noveron *et al.*, 2003 ▸), with bis­(hexa­fluoro­acetyl­acetonato) as an ancillary ligand.

## Synthesis and crystallization   

The heterocyclic nitro­gen ligand (H_2_ *L* ^1^) was prepared using a modified literature procedure (Qin *et al.*, 2003 ▸; Li *et al.*, 2012 ▸). In the preparation of complex (I), H_2_ *L* ^1^ (0.1 mmol, 0.032 g) in *N*,*N*′-di­methyl­formamide solution (4 mL) was gradually added to MnSO_4_^.^H_2_O (0.1 mmol, 0.017 g) in a mixed solution (3 mL, water–methanol *v*/*v* = 1/3). After standing for 5 min, the suspension was filtered and the filtrate was kept at room temperature in the dark. One week later, colourless single crystals suitable for X-ray diffraction were obtained. Complex (II) was prepared with the same procedure employed for (I) except that CdSO_4_^.^8/3H_2_O (0.1 mmol, 0.026 g) was used instead of MnSO_4_^.^H_2_O.

## Refinement   

Crystal data, data collection and structure refinement details are summarized in Table 5 ▸. Water H atoms were located in a difference-Fourier map and freely refined. All other H atoms were positioned gemetrically and refined using a riding model with bond lengths of 0.93 Å (C—H, aromatic), 0.83 Å (N—H) and 0.85 Å (O—H), and with *U*_iso_(H) = 1.2–1.5*U*_eq_(C/N/O).

## Supplementary Material

Crystal structure: contains datablock(s) I, II. DOI: 10.1107/S2056989018007351/cq2024sup1.cif

Structure factors: contains datablock(s) I. DOI: 10.1107/S2056989018007351/cq2024Isup2.hkl

Structure factors: contains datablock(s) II. DOI: 10.1107/S2056989018007351/cq2024IIsup3.hkl

CCDC references: 1822492, 1822490

Additional supporting information:  crystallographic information; 3D view; checkCIF report

## Figures and Tables

**Figure 6:**
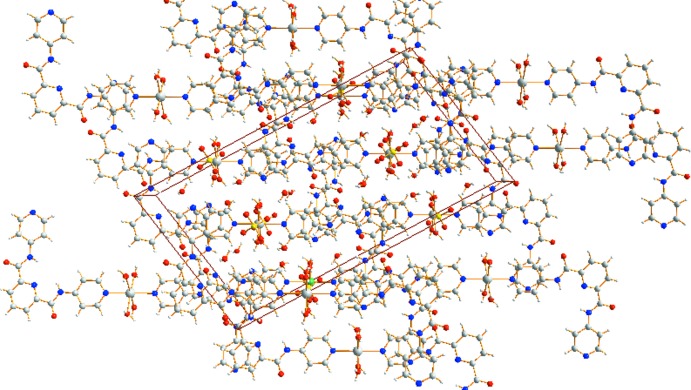
The mol­ecular packing of (II) viewed along the *b* axis.

**Table 1:** Selected geometric parameters (Å, °) for (I)

Mn1—O10	2.1491 (15)	Mn1—N1	2.2208 (17)
Mn1—O6	2.1502 (17)	Mn1—O5	2.2298 (16)
Mn1—O4	2.1938 (17)	Mn1—O3	2.2333 (17)
			
O6—Mn1—O4	175.13 (6)	O6—Mn1—O3	85.79 (7)
O10—Mn1—N1	177.74 (6)	O4—Mn1—O3	90.90 (7)
O6—Mn1—O5	91.71 (6)	O5—Mn1—O3	175.51 (6)
O4—Mn1—O5	91.35 (7)		

**Table 2:** Selected geometric parameters (Å, °) for (II)

Cd1—N1	2.275 (3)	Cd1—O6	2.371 (3)
Cd1—O5	2.334 (2)		
			
N1^i^—Cd1—N1	177.40 (15)	O5—Cd1—O6	175.09 (10)
N1—Cd1—O5^i^	90.92 (10)	N1^i^—Cd1—O6^i^	90.88 (10)
N1—Cd1—O5	91.04 (10)	N1—Cd1—O6^i^	87.31 (10)
O5^i^—Cd1—O5	82.63 (14)	O5—Cd1—O6^i^	92.82 (10)
N1—Cd1—O6	90.88 (10)	O6—Cd1—O6^i^	91.79 (15)

Symmetry code: (i) 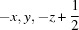.

**Table 3:** Hydrogen-bond geometry (Å, °) for (I)

*D*—H⋯*A*	*D*—H	H⋯*A*	*D*⋯*A*	*D*—H⋯*A*
O3—H3⋯O10^i^	0.82	2.34	3.057 (3)	147
O4—H4⋯O8	0.82	1.98	2.754 (2)	158
O5—H5⋯O8^ii^	0.82	1.98	2.774 (2)	163
O6—H6⋯O7^i^	0.82	2.02	2.834 (2)	170
O12—H18⋯O9^iii^	0.83 (3)	1.97 (3)	2.772 (3)	165 (4)
O12—H19⋯O7^iv^	0.81 (4)	2.47 (4)	3.169 (3)	145 (3)
O12—H19⋯O9^iv^	0.81 (4)	2.43 (4)	3.128 (3)	145 (3)
N2—H20⋯N5	0.85 (3)	2.34 (2)	2.733 (2)	109.0 (18)
N2—H20⋯O11^v^	0.85 (3)	2.11 (3)	2.910 (3)	157 (2)
O6—H21⋯N4^vi^	0.85 (3)	1.84 (3)	2.686 (3)	173 (3)
O5—H22⋯O12^vi^	0.81 (3)	2.08 (3)	2.883 (3)	171 (3)
O4—H23⋯O9^ii^	0.83 (3)	2.02 (4)	2.828 (2)	168 (3)
O3—H24⋯O12^vii^	0.85 (4)	2.32 (4)	3.165 (3)	171 (4)
N3—H25⋯N5	0.85 (3)	2.28 (2)	2.709 (2)	111.4 (16)
N3—H25⋯O11^v^	0.85 (3)	2.24 (2)	2.979 (2)	147 (3)
O11—H26⋯O7^iii^	0.86 (3)	1.92 (3)	2.774 (2)	176 (3)
O11—H27⋯O1^viii^	0.78 (3)	2.08 (3)	2.843 (2)	168 (3)

Symmetry codes: (i) 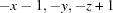; (ii) 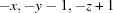; (iii) 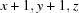; (iv) 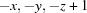; (v) 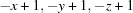; (vi) 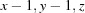; (vii) 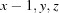; (viii) 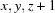.

**Table 4:** Hydrogen-bond geometry (Å, °) for (II)

*D*—H⋯*A*	*D*—H	H⋯*A*	*D*⋯*A*	*D*—H⋯*A*
N2—H2*B*⋯O7^ii^	0.84 (4)	2.17 (4)	2.958 (4)	156 (4)
N2—H2*B*⋯N3	0.84 (4)	2.35 (4)	2.736 (4)	109 (3)
N4—H4*B*⋯O7^ii^	0.83 (4)	2.29 (4)	3.030 (4)	150 (4)
N4—H4*B*⋯N3	0.83 (4)	2.29 (4)	2.698 (4)	111 (3)
O5—H5*A*⋯N5^iii^	0.80 (4)	1.91 (4)	2.704 (4)	174 (4)
O5—H5*B*⋯O3^iii^	0.79 (4)	2.02 (4)	2.811 (4)	172 (4)
O6—H6*A*⋯O4^iv^	0.82 (5)	1.98 (5)	2.775 (6)	161 (6)
O6—H6*B*⋯O8^v^	0.84 (6)	2.04 (6)	2.872 (4)	173 (6)
O7—H7*A*⋯O3^vi^	0.86 (5)	1.93 (5)	2.784 (4)	174 (4)
O7—H7*B*⋯O2^vii^	0.82 (5)	2.13 (5)	2.912 (4)	159 (5)
O8—H8*B*⋯O5^viii^	0.80	2.33	3.052 (4)	151
O8—H8*C*⋯O4^vi^	0.80	2.53	3.233 (6)	147
O8—H8*C*⋯O3^ix^	0.80	2.36	3.085 (4)	152

Symmetry codes: (ii) 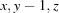; (iii) 
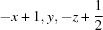
; (iv) 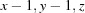; (v) 
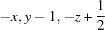
; (vi) 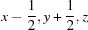; (vii) 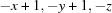; (viii) 
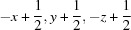
; (ix) 
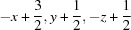
.

**Table 5:** Experimental details

	(I)	(II)
Crystal data		
Chemical formula	[Mn(SO_4_)(C_17_H_13_N_5_O_2_)(H_2_O)_4_·2H_2_O	[Cd(C_17_H_13_N_5_O_2_)_2_(H_2_O)_4_](SO_4_)·4H_2_O
*M*_r_	578.42	991.23
Crystal system, space group	Triclinic, *P* 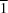	Monoclinic, *C*2/*c*
Temperature (K)	293	293
*a*, *b*, *c* (Å)	8.9333 (18), 8.9998 (18), 15.949 (3)	14.706 (3), 10.157 (2), 27.293 (6)
α, β, γ (°)	78.92 (3), 81.04 (3), 68.43 (3)	90, 99.52 (3), 90
*V* (Å^3^)	1165.1 (5)	4020.6 (14)
*Z*	2	4
Radiation type	Mo *K*α	Mo *K*α
μ (mm^−1^)	0.73	0.68
Crystal size (mm)	0.20 × 0.20 × 0.20	0.20 × 0.20 × 0.20

Data collection		
Diffractometer	Rigaku Saturn724	Rigaku Saturn724
Absorption correction	Multi-scan (*CrystalClear*; Rigaku/MSC, 2006 ▸)	Multi-scan (*CrystalClear*; Rigaku/MSC, 2006 ▸)
*T*_min_, *T*_max_	0.939, 1.000	0.780, 1.000
No. of measured, independent and observed [*I* > 2σ(*I*)] reflections	14157, 5299, 4440	22754, 4592, 4261
*R*_int_	0.024	0.061
(sin θ/λ)_max_ (Å^−1^)	0.650	0.650

Refinement		
*R*[*F* ^2^ > 2σ(*F* ^2^)], *wR*(*F* ^2^), *S*	0.037, 0.092, 1.07	0.051, 0.128, 1.14
No. of reflections	5299	4592
No. of parameters	369	313
H-atom treatment	H atoms treated by a mixture of independent and constrained refinement	H atoms treated by a mixture of independent and constrained refinement
Δρ_max_, Δρ_min_ (e Å^−3^)	0.24, −0.45	0.81, −0.85

Computer programs: *CrystalClear* (Rigaku/MSC, 2006 ▸), *SHELXT2014/7* (Sheldrick, 2015*a* ▸) and *SHELXL2014/7* (Sheldrick, 2015*b* ▸).

## supplementary crystallographic information

### Tetraaqua[N,N'-bis(pyridin-4-yl)pyridine-2,6-dicarboxamide]sulfatomanganese(II) dihydrate (I) Crystal data

**Table:**

[Mn(SO_4_)(C_17_H_13_N_5_O_2_)(H_2_O)_4_]·2H_2_O	*Z* = 2
*M**_r_* = 578.42	*F*(000) = 598
Triclinic, *P*1	*D*_x_ = 1.649 Mg m^−^^3^
*a* = 8.9333 (18) Å	Mo *K*α radiation, λ = 0.71073 Å
*b* = 8.9998 (18) Å	Cell parameters from 3219 reflections
*c* = 15.949 (3) Å	θ = 3.3–27.5°
α = 78.92 (3)°	µ = 0.73 mm^−^^1^
β = 81.04 (3)°	*T* = 293 K
γ = 68.43 (3)°	Prism, colorless
*V* = 1165.1 (5) Å^3^	0.20 × 0.20 × 0.20 mm

### Tetraaqua[N,N'-bis(pyridin-4-yl)pyridine-2,6-dicarboxamide]sulfatomanganese(II) dihydrate (I) Data collection

**Table:**

Rigaku Saturn724 diffractometer	4440 reflections with *I* > 2σ(*I*)
Detector resolution: 28.5714 pixels mm^-1^	*R*_int_ = 0.024
dtprofit.ref scans	θ_max_ = 27.5°, θ_min_ = 3.3°
Absorption correction: multi-scan (CrystalClear; Rigaku/MSC, 2006)	*h* = −11→11
*T*_min_ = 0.939, *T*_max_ = 1.000	*k* = −11→11
14157 measured reflections	*l* = −20→20
5299 independent reflections	

### Tetraaqua[N,N'-bis(pyridin-4-yl)pyridine-2,6-dicarboxamide]sulfatomanganese(II) dihydrate (I) Refinement

**Table:**

Refinement on *F*^2^	0 restraints
Least-squares matrix: full	Hydrogen site location: mixed
*R*[*F*^2^ > 2σ(*F*^2^)] = 0.037	H atoms treated by a mixture of independent and constrained refinement
*wR*(*F*^2^) = 0.092	*w* = 1/[σ^2^(*F*_o_^2^) + (0.0475*P*)^2^ + 0.1434*P*] where *P* = (*F*_o_^2^ + 2*F*_c_^2^)/3
*S* = 1.07	(Δ/σ)_max_ < 0.001
5299 reflections	Δρ_max_ = 0.24 e Å^−^^3^
369 parameters	Δρ_min_ = −0.45 e Å^−^^3^

### Tetraaqua[N,N'-bis(pyridin-4-yl)pyridine-2,6-dicarboxamide]sulfatomanganese(II) dihydrate (I) Special details

**Table:**

Geometry. All esds (except the esd in the dihedral angle between two l.s. planes) are estimated using the full covariance matrix. The cell esds are taken into account individually in the estimation of esds in distances, angles and torsion angles; correlations between esds in cell parameters are only used when they are defined by crystal symmetry. An approximate (isotropic) treatment of cell esds is used for estimating esds involving l.s. planes.

### Tetraaqua[N,N'-bis(pyridin-4-yl)pyridine-2,6-dicarboxamide]sulfatomanganese(II) dihydrate (I) Fractional atomic coordinates and isotropic or equivalent isotropic displacement parameters (Å^2^)

**Table:**

	*x*	*y*	*z*	*U*_iso_*/*U*_eq_	
C1	0.2351 (2)	0.4933 (2)	−0.18307 (12)	0.0332 (4)	
H1	0.2377	0.5031	−0.2424	0.040*	
C2	0.1757 (2)	0.3842 (2)	−0.13011 (12)	0.0323 (4)	
H2	0.1351	0.3207	−0.1530	0.039*	
C3	0.1769 (2)	0.3699 (2)	−0.04191 (11)	0.0254 (4)	
C4	0.2824 (2)	0.5697 (2)	−0.05814 (11)	0.0251 (4)	
C5	0.2907 (2)	0.5875 (2)	−0.14691 (12)	0.0297 (4)	
H5A	0.3329	0.6616	−0.1811	0.036*	
C6	0.3359 (2)	0.6775 (2)	−0.01818 (12)	0.0271 (4)	
C7	0.1178 (2)	0.2448 (2)	0.01474 (12)	0.0290 (4)	
C8	0.0623 (2)	0.1421 (2)	0.16695 (12)	0.0263 (4)	
C9	0.0734 (2)	0.1556 (2)	0.25115 (12)	0.0321 (4)	
H9	0.1269	0.2202	0.2620	0.039*	
C10	0.0053 (3)	0.0734 (3)	0.31810 (13)	0.0356 (5)	
H10	0.0158	0.0829	0.3737	0.043*	
C11	−0.0834 (2)	−0.0329 (2)	0.22587 (12)	0.0331 (4)	
H11	−0.1387	−0.0971	0.2168	0.040*	
C12	−0.0160 (2)	0.0415 (2)	0.15486 (12)	0.0310 (4)	
H12	−0.0228	0.0248	0.1000	0.037*	
C13	0.3595 (2)	0.7410 (2)	0.12187 (11)	0.0255 (4)	
C14	0.3298 (2)	0.7027 (2)	0.21023 (12)	0.0315 (4)	
H14	0.2766	0.6302	0.2325	0.038*	
C15	0.3801 (3)	0.7733 (2)	0.26411 (13)	0.0354 (5)	
H15	0.3585	0.7473	0.3230	0.042*	
C16	0.4822 (2)	0.9162 (2)	0.15173 (13)	0.0345 (4)	
H16	0.5342	0.9904	0.1314	0.041*	
C17	0.4350 (2)	0.8534 (2)	0.09234 (12)	0.0314 (4)	
H17	0.4535	0.8856	0.0339	0.038*	
H18	0.663 (4)	0.456 (4)	0.455 (2)	0.092 (12)*	
H19	0.587 (4)	0.454 (4)	0.389 (2)	0.086 (12)*	
H20	0.167 (3)	0.295 (3)	0.1157 (15)	0.048 (7)*	
H21	−0.463 (4)	−0.051 (3)	0.3149 (18)	0.070 (9)*	
H22	−0.202 (4)	−0.400 (3)	0.3595 (18)	0.065 (9)*	
H23	0.090 (4)	−0.300 (4)	0.4613 (18)	0.067 (9)*	
H24	−0.293 (5)	0.175 (5)	0.462 (3)	0.138 (17)*	
H25	0.278 (3)	0.588 (3)	0.0925 (13)	0.037 (6)*	
H26	0.645 (3)	0.661 (3)	0.7711 (19)	0.065 (9)*	
H27	0.593 (4)	0.694 (3)	0.8471 (19)	0.062 (10)*	
Mn1	−0.21144 (3)	−0.13074 (3)	0.41352 (2)	0.02613 (9)	
N1	−0.0756 (2)	−0.0201 (2)	0.30737 (10)	0.0328 (4)	
N2	0.1244 (2)	0.23492 (19)	0.10064 (10)	0.0279 (3)	
N3	0.3141 (2)	0.66182 (19)	0.06822 (10)	0.0286 (3)	
N4	0.4582 (2)	0.8772 (2)	0.23662 (11)	0.0356 (4)	
N5	0.22775 (18)	0.46192 (17)	−0.00561 (9)	0.0248 (3)	
O1	0.39470 (19)	0.77213 (17)	−0.06388 (9)	0.0391 (3)	
O2	0.0668 (2)	0.16229 (19)	−0.01660 (9)	0.0472 (4)	
O3	−0.2975 (2)	0.0822 (2)	0.48362 (12)	0.0453 (4)	
H3	−0.3920	0.0995	0.5027	0.068*	
O4	0.00523 (19)	−0.23999 (19)	0.48356 (10)	0.0390 (3)	
H4	−0.0150	−0.2930	0.5288	0.059*	
O5	−0.14334 (18)	−0.34701 (17)	0.34781 (10)	0.0352 (3)	
H5	−0.0516	−0.4073	0.3579	0.053*	
O6	−0.43556 (19)	−0.01988 (18)	0.35520 (11)	0.0434 (4)	
H6	−0.4679	0.0780	0.3552	0.065*	
O7	−0.42592 (18)	−0.32142 (18)	0.66102 (9)	0.0429 (4)	
O8	−0.14065 (18)	−0.39726 (18)	0.61734 (10)	0.0418 (4)	
O9	−0.29838 (19)	−0.52446 (17)	0.56986 (10)	0.0423 (4)	
O10	−0.33597 (17)	−0.24703 (16)	0.51556 (9)	0.0354 (3)	
O11	0.6732 (2)	0.6493 (2)	0.82167 (11)	0.0396 (4)	
O12	0.6731 (2)	0.4413 (3)	0.40463 (14)	0.0622 (5)	
S2	−0.29885 (5)	−0.37332 (5)	0.59117 (3)	0.02647 (12)	

### Tetraaqua[N,N'-bis(pyridin-4-yl)pyridine-2,6-dicarboxamide]sulfatomanganese(II) dihydrate (I) Atomic displacement parameters (Å^2^)

**Table:**

	*U*^11^	*U*^22^	*U*^33^	*U*^12^	*U*^13^	*U*^23^
C1	0.0400 (11)	0.0386 (11)	0.0209 (9)	−0.0124 (9)	−0.0036 (8)	−0.0061 (8)
C2	0.0382 (11)	0.0331 (10)	0.0289 (10)	−0.0133 (9)	−0.0051 (8)	−0.0085 (8)
C3	0.0249 (9)	0.0264 (9)	0.0261 (9)	−0.0091 (7)	−0.0029 (7)	−0.0056 (7)
C4	0.0240 (9)	0.0262 (9)	0.0249 (9)	−0.0090 (7)	−0.0022 (7)	−0.0031 (7)
C5	0.0322 (10)	0.0312 (10)	0.0245 (9)	−0.0121 (8)	−0.0010 (8)	−0.0006 (7)
C6	0.0284 (9)	0.0265 (9)	0.0268 (9)	−0.0116 (7)	−0.0020 (7)	−0.0011 (7)
C7	0.0322 (10)	0.0277 (9)	0.0288 (10)	−0.0125 (8)	−0.0012 (8)	−0.0053 (8)
C8	0.0267 (9)	0.0232 (9)	0.0286 (9)	−0.0092 (7)	−0.0031 (7)	−0.0018 (7)
C9	0.0368 (11)	0.0374 (11)	0.0308 (10)	−0.0223 (9)	−0.0039 (8)	−0.0055 (8)
C10	0.0436 (12)	0.0434 (12)	0.0268 (10)	−0.0241 (10)	−0.0015 (8)	−0.0048 (8)
C11	0.0409 (11)	0.0317 (10)	0.0333 (11)	−0.0212 (9)	−0.0024 (9)	−0.0038 (8)
C12	0.0391 (11)	0.0316 (10)	0.0279 (10)	−0.0186 (9)	−0.0028 (8)	−0.0048 (8)
C13	0.0279 (9)	0.0236 (9)	0.0259 (9)	−0.0094 (7)	−0.0044 (7)	−0.0035 (7)
C14	0.0400 (11)	0.0300 (10)	0.0277 (10)	−0.0178 (9)	0.0001 (8)	−0.0032 (8)
C15	0.0480 (12)	0.0325 (11)	0.0270 (10)	−0.0152 (9)	−0.0044 (9)	−0.0045 (8)
C16	0.0409 (11)	0.0296 (10)	0.0388 (11)	−0.0186 (9)	−0.0038 (9)	−0.0058 (8)
C17	0.0398 (11)	0.0304 (10)	0.0263 (10)	−0.0167 (9)	−0.0012 (8)	−0.0021 (8)
Mn1	0.02740 (16)	0.02532 (16)	0.02546 (16)	−0.01036 (12)	−0.00155 (11)	−0.00162 (11)
N1	0.0377 (9)	0.0340 (9)	0.0318 (9)	−0.0209 (7)	0.0006 (7)	−0.0028 (7)
N2	0.0348 (9)	0.0297 (8)	0.0259 (8)	−0.0191 (7)	−0.0026 (7)	−0.0038 (6)
N3	0.0365 (9)	0.0295 (9)	0.0255 (8)	−0.0197 (7)	−0.0016 (7)	−0.0019 (6)
N4	0.0431 (10)	0.0317 (9)	0.0358 (9)	−0.0143 (8)	−0.0077 (8)	−0.0083 (7)
N5	0.0262 (8)	0.0253 (8)	0.0242 (8)	−0.0106 (6)	−0.0022 (6)	−0.0036 (6)
O1	0.0562 (10)	0.0421 (8)	0.0287 (7)	−0.0321 (7)	0.0009 (6)	−0.0014 (6)
O2	0.0780 (12)	0.0517 (9)	0.0327 (8)	−0.0456 (9)	−0.0039 (8)	−0.0092 (7)
O3	0.0421 (9)	0.0416 (9)	0.0570 (11)	−0.0162 (7)	0.0034 (8)	−0.0218 (8)
O4	0.0335 (8)	0.0401 (9)	0.0411 (9)	−0.0111 (7)	−0.0072 (7)	−0.0007 (7)
O5	0.0352 (8)	0.0310 (8)	0.0403 (8)	−0.0115 (6)	−0.0031 (6)	−0.0079 (6)
O6	0.0481 (9)	0.0311 (8)	0.0518 (10)	−0.0030 (7)	−0.0267 (8)	−0.0118 (7)
O7	0.0436 (9)	0.0448 (9)	0.0263 (7)	−0.0043 (7)	0.0058 (6)	−0.0022 (6)
O8	0.0380 (8)	0.0427 (9)	0.0447 (9)	−0.0136 (7)	−0.0124 (7)	−0.0007 (7)
O9	0.0523 (9)	0.0318 (8)	0.0468 (9)	−0.0186 (7)	−0.0056 (7)	−0.0062 (6)
O10	0.0348 (8)	0.0363 (8)	0.0307 (7)	−0.0134 (6)	−0.0024 (6)	0.0072 (6)
O11	0.0480 (10)	0.0516 (10)	0.0280 (8)	−0.0280 (8)	−0.0045 (7)	−0.0044 (7)
O12	0.0493 (11)	0.1032 (17)	0.0448 (11)	−0.0374 (11)	−0.0040 (9)	−0.0148 (11)
S2	0.0293 (2)	0.0244 (2)	0.0232 (2)	−0.00813 (19)	−0.00086 (18)	−0.00150 (17)

### Tetraaqua[N,N'-bis(pyridin-4-yl)pyridine-2,6-dicarboxamide]sulfatomanganese(II) dihydrate (I) Geometric parameters (Å, º)

**Table:**

C1—C2	1.372 (3)	C15—N4	1.334 (3)
C1—C5	1.373 (3)	C15—H15	0.9300
C1—H1	0.9300	C16—N4	1.336 (3)
C2—C3	1.389 (3)	C16—C17	1.378 (3)
C2—H2	0.9300	C16—H16	0.9300
C3—N5	1.331 (2)	C17—H17	0.9300
C3—C7	1.503 (3)	Mn1—O10	2.1491 (15)
C4—N5	1.338 (2)	Mn1—O6	2.1502 (17)
C4—C5	1.387 (2)	Mn1—O4	2.1938 (17)
C4—C6	1.500 (2)	Mn1—N1	2.2208 (17)
C5—H5A	0.9300	Mn1—O5	2.2298 (16)
C6—O1	1.226 (2)	Mn1—O3	2.2333 (17)
C6—N3	1.348 (2)	N2—H20	0.85 (2)
C7—O2	1.213 (2)	N3—H25	0.84 (2)
C7—N2	1.365 (2)	O3—H24	0.85 (4)
C8—C12	1.386 (3)	O3—H3	0.8200
C8—C9	1.393 (3)	O4—H23	0.83 (3)
C8—N2	1.394 (2)	O4—H4	0.8200
C9—C10	1.372 (3)	O5—H22	0.82 (3)
C9—H9	0.9300	O5—H5	0.8200
C10—N1	1.342 (2)	O6—H21	0.85 (3)
C10—H10	0.9300	O6—H6	0.8200
C11—N1	1.341 (3)	O7—S2	1.4711 (15)
C11—C12	1.373 (3)	O8—S2	1.4640 (15)
C11—H11	0.9300	O9—S2	1.4624 (14)
C12—H12	0.9300	O10—S2	1.4753 (14)
C13—C17	1.386 (2)	O11—H26	0.86 (3)
C13—C14	1.392 (3)	O11—H27	0.77 (3)
C13—N3	1.399 (2)	O12—H18	0.83 (4)
C14—C15	1.372 (3)	O12—H19	0.81 (4)
C14—H14	0.9300		
			
C2—C1—C5	118.78 (17)	C16—C17—H17	120.8
C2—C1—H1	120.6	C13—C17—H17	120.8
C5—C1—H1	120.6	O10—Mn1—O6	87.71 (7)
C1—C2—C3	119.17 (18)	O10—Mn1—O4	88.59 (6)
C1—C2—H2	120.4	O6—Mn1—O4	175.13 (6)
C3—C2—H2	120.4	O10—Mn1—N1	177.74 (6)
N5—C3—C2	122.91 (17)	O6—Mn1—N1	93.60 (7)
N5—C3—C7	118.79 (16)	O4—Mn1—N1	90.19 (7)
C2—C3—C7	118.31 (16)	O10—Mn1—O5	88.16 (6)
N5—C4—C5	123.57 (16)	O6—Mn1—O5	91.71 (6)
N5—C4—C6	117.74 (15)	O4—Mn1—O5	91.35 (7)
C5—C4—C6	118.68 (16)	N1—Mn1—O5	89.96 (6)
C1—C5—C4	118.43 (18)	O10—Mn1—O3	88.01 (7)
C1—C5—H5A	120.8	O6—Mn1—O3	85.79 (7)
C4—C5—H5A	120.8	O4—Mn1—O3	90.90 (7)
O1—C6—N3	124.43 (17)	N1—Mn1—O3	93.91 (7)
O1—C6—C4	119.88 (16)	O5—Mn1—O3	175.51 (6)
N3—C6—C4	115.69 (16)	C11—N1—C10	115.82 (17)
O2—C7—N2	124.42 (18)	C11—N1—Mn1	119.92 (13)
O2—C7—C3	120.07 (17)	C10—N1—Mn1	124.00 (13)
N2—C7—C3	115.50 (16)	C7—N2—C8	126.96 (16)
C12—C8—C9	117.44 (17)	C7—N2—H20	116.9 (16)
C12—C8—N2	124.29 (17)	C8—N2—H20	116.0 (16)
C9—C8—N2	118.22 (16)	C6—N3—C13	127.71 (16)
C10—C9—C8	119.91 (17)	C6—N3—H25	115.3 (14)
C10—C9—H9	120.0	C13—N3—H25	116.6 (14)
C8—C9—H9	120.0	C15—N4—C16	116.70 (17)
N1—C10—C9	123.33 (18)	C3—N5—C4	117.08 (15)
N1—C10—H10	118.3	Mn1—O3—H24	125 (3)
C9—C10—H10	118.3	Mn1—O3—H3	109.5
N1—C11—C12	125.09 (18)	H24—O3—H3	105.1
N1—C11—H11	117.5	Mn1—O4—H23	120 (2)
C12—C11—H11	117.5	Mn1—O4—H4	109.5
C11—C12—C8	118.35 (18)	H23—O4—H4	105.3
C11—C12—H12	120.8	Mn1—O5—H22	117 (2)
C8—C12—H12	120.8	Mn1—O5—H5	109.5
C17—C13—C14	117.86 (17)	H22—O5—H5	107.8
C17—C13—N3	123.86 (17)	Mn1—O6—H21	126.4 (19)
C14—C13—N3	118.27 (16)	Mn1—O6—H6	109.5
C15—C14—C13	119.23 (18)	H21—O6—H6	116.0
C15—C14—H14	120.4	S2—O10—Mn1	139.12 (9)
C13—C14—H14	120.4	H26—O11—H27	103 (3)
N4—C15—C14	123.52 (19)	H18—O12—H19	111 (3)
N4—C15—H15	118.2	O9—S2—O8	109.64 (9)
C14—C15—H15	118.2	O9—S2—O7	109.31 (10)
N4—C16—C17	124.19 (18)	O8—S2—O7	110.22 (9)
N4—C16—H16	117.9	O9—S2—O10	109.28 (9)
C17—C16—H16	117.9	O8—S2—O10	110.20 (9)
C16—C17—C13	118.42 (18)	O7—S2—O10	108.16 (9)
			
C5—C1—C2—C3	−1.4 (3)	C14—C13—C17—C16	2.5 (3)
C1—C2—C3—N5	2.6 (3)	N3—C13—C17—C16	−176.26 (18)
C1—C2—C3—C7	−177.44 (18)	C12—C11—N1—C10	−0.1 (3)
C2—C1—C5—C4	−0.8 (3)	C12—C11—N1—Mn1	174.26 (16)
N5—C4—C5—C1	2.1 (3)	C9—C10—N1—C11	1.7 (3)
C6—C4—C5—C1	−177.26 (17)	C9—C10—N1—Mn1	−172.42 (16)
N5—C4—C6—O1	176.21 (17)	O2—C7—N2—C8	−6.2 (3)
C5—C4—C6—O1	−4.4 (3)	C3—C7—N2—C8	172.84 (17)
N5—C4—C6—N3	−3.9 (2)	C12—C8—N2—C7	0.8 (3)
C5—C4—C6—N3	175.47 (17)	C9—C8—N2—C7	−176.46 (18)
N5—C3—C7—O2	178.03 (18)	O1—C6—N3—C13	−3.4 (3)
C2—C3—C7—O2	−1.9 (3)	C4—C6—N3—C13	176.79 (17)
N5—C3—C7—N2	−1.1 (3)	C17—C13—N3—C6	0.8 (3)
C2—C3—C7—N2	179.01 (17)	C14—C13—N3—C6	−177.94 (19)
C12—C8—C9—C10	−1.3 (3)	C14—C15—N4—C16	2.2 (3)
N2—C8—C9—C10	176.22 (18)	C17—C16—N4—C15	−1.5 (3)
C8—C9—C10—N1	−1.0 (3)	C2—C3—N5—C4	−1.4 (3)
N1—C11—C12—C8	−2.1 (3)	C7—C3—N5—C4	178.70 (16)
C9—C8—C12—C11	2.7 (3)	C5—C4—N5—C3	−1.0 (3)
N2—C8—C12—C11	−174.63 (18)	C6—C4—N5—C3	178.35 (15)
C17—C13—C14—C15	−1.9 (3)	Mn1—O10—S2—O9	−100.05 (14)
N3—C13—C14—C15	176.98 (18)	Mn1—O10—S2—O8	20.49 (16)
C13—C14—C15—N4	−0.6 (3)	Mn1—O10—S2—O7	141.04 (13)
N4—C16—C17—C13	−0.8 (3)		

### Tetraaqua[N,N'-bis(pyridin-4-yl)pyridine-2,6-dicarboxamide]sulfatomanganese(II) dihydrate (I) Hydrogen-bond geometry (Å, º)

**Table:**

*D*—H···*A*	*D*—H	H···*A*	*D*···*A*	*D*—H···*A*
O3—H3···O10^i^	0.82	2.34	3.057 (3)	147
O4—H4···O8	0.82	1.98	2.754 (2)	158
O5—H5···O8^ii^	0.82	1.98	2.774 (2)	163
O6—H6···O7^i^	0.82	2.02	2.834 (2)	170
O12—H18···O9^iii^	0.83 (3)	1.97 (3)	2.772 (3)	165 (4)
O12—H19···O7^iv^	0.81 (4)	2.47 (4)	3.169 (3)	145 (3)
O12—H19···O9^iv^	0.81 (4)	2.43 (4)	3.128 (3)	145 (3)
N2—H20···N5	0.85 (3)	2.34 (2)	2.733 (2)	109.0 (18)
N2—H20···O11^v^	0.85 (3)	2.11 (3)	2.910 (3)	157 (2)
O6—H21···N4^vi^	0.85 (3)	1.84 (3)	2.686 (3)	173 (3)
O5—H22···O12^vi^	0.81 (3)	2.08 (3)	2.883 (3)	171 (3)
O4—H23···O9^ii^	0.83 (3)	2.02 (4)	2.828 (2)	168 (3)
O3—H24···O12^vii^	0.85 (4)	2.32 (4)	3.165 (3)	171 (4)
N3—H25···N5	0.85 (3)	2.28 (2)	2.709 (2)	111.4 (16)
N3—H25···O11^v^	0.85 (3)	2.24 (2)	2.979 (2)	147 (3)
O11—H26···O7^iii^	0.86 (3)	1.92 (3)	2.774 (2)	176 (3)
O11—H27···O1^viii^	0.78 (3)	2.08 (3)	2.843 (2)	168 (3)

Symmetry codes: (i) −*x*−1, −*y*, −*z*+1; (ii) −*x*, −*y*−1, −*z*+1; (iii) *x*+1, *y*+1, *z*; (iv) −*x*, −*y*, −*z*+1; (v) −*x*+1, −*y*+1, −*z*+1; (vi) *x*−1, *y*−1, *z*; (vii) *x*−1, *y*, *z*; (viii) *x*, *y*, *z*+1.

### Tetraaquabis[N,N'-bis(pyridin-4-yl)pyridine-2,6-\ dicarboxamide]cadmium(II) sulfate tetrahydrate (II) Crystal data

**Table:**

[Cd(C_17_H_13_N_5_O_2_)_2_(H_2_O)_4_]SO_4_·4H_2_O	*F*(000) = 2032
*M**_r_* = 991.23	*D*_x_ = 1.638 Mg m^−^^3^
Monoclinic, *C*2/*c*	Mo *K*α radiation, λ = 0.71073 Å
*a* = 14.706 (3) Å	Cell parameters from 5365 reflections
*b* = 10.157 (2) Å	θ = 3.0–27.5°
*c* = 27.293 (6) Å	µ = 0.68 mm^−^^1^
β = 99.52 (3)°	*T* = 293 K
*V* = 4020.6 (14) Å^3^	Prism, colorless
*Z* = 4	0.20 × 0.20 × 0.20 mm

### Tetraaquabis[N,N'-bis(pyridin-4-yl)pyridine-2,6-\ dicarboxamide]cadmium(II) sulfate tetrahydrate (II) Data collection

**Table:**

Rigaku Saturn724 diffractometer	4261 reflections with *I* > 2σ(*I*)
Detector resolution: 28.5714 pixels mm^-1^	*R*_int_ = 0.061
dtprofit.ref scans	θ_max_ = 27.5°, θ_min_ = 3.0°
Absorption correction: multi-scan (CrystalClear; Rigaku/MSC, 2006)	*h* = −19→19
*T*_min_ = 0.780, *T*_max_ = 1.000	*k* = −13→13
22754 measured reflections	*l* = −35→32
4592 independent reflections	

### Tetraaquabis[N,N'-bis(pyridin-4-yl)pyridine-2,6-\ dicarboxamide]cadmium(II) sulfate tetrahydrate (II) Refinement

**Table:**

Refinement on *F*^2^	0 restraints
Least-squares matrix: full	Hydrogen site location: mixed
*R*[*F*^2^ > 2σ(*F*^2^)] = 0.051	H atoms treated by a mixture of independent and constrained refinement
*wR*(*F*^2^) = 0.128	*w* = 1/[σ^2^(*F*_o_^2^) + (0.0648*P*)^2^ + 3.9753*P*] where *P* = (*F*_o_^2^ + 2*F*_c_^2^)/3
*S* = 1.14	(Δ/σ)_max_ < 0.001
4592 reflections	Δρ_max_ = 0.81 e Å^−^^3^
313 parameters	Δρ_min_ = −0.85 e Å^−^^3^

### Tetraaquabis[N,N'-bis(pyridin-4-yl)pyridine-2,6-\ dicarboxamide]cadmium(II) sulfate tetrahydrate (II) Special details

**Table:**

Geometry. All esds (except the esd in the dihedral angle between two l.s. planes) are estimated using the full covariance matrix. The cell esds are taken into account individually in the estimation of esds in distances, angles and torsion angles; correlations between esds in cell parameters are only used when they are defined by crystal symmetry. An approximate (isotropic) treatment of cell esds is used for estimating esds involving l.s. planes.

### Tetraaquabis[N,N'-bis(pyridin-4-yl)pyridine-2,6-\ dicarboxamide]cadmium(II) sulfate tetrahydrate (II) Fractional atomic coordinates and isotropic or equivalent isotropic displacement parameters (Å^2^)

**Table:**

	*x*	*y*	*z*	*U*_iso_*/*U*_eq_	
C1	0.1669 (2)	0.0681 (3)	0.18881 (12)	0.0366 (7)	
H1A	0.2000	0.0777	0.2207	0.044*	
C2	0.0297 (2)	0.0417 (4)	0.13698 (13)	0.0385 (7)	
H2A	−0.0341	0.0331	0.1326	0.046*	
C3	0.0710 (2)	0.0386 (3)	0.09526 (12)	0.0355 (7)	
H3A	0.0362	0.0267	0.0639	0.043*	
C4	0.2149 (2)	0.0669 (3)	0.14971 (12)	0.0343 (7)	
H4A	0.2788	0.0747	0.1553	0.041*	
C5	0.1666 (2)	0.0538 (3)	0.10146 (11)	0.0296 (6)	
C6	0.1819 (2)	0.0643 (3)	0.01320 (11)	0.0319 (6)	
C7	0.2508 (2)	0.0910 (3)	−0.02056 (11)	0.0292 (6)	
C8	0.2192 (2)	0.0944 (3)	−0.07171 (11)	0.0356 (7)	
H8A	0.1570	0.0823	−0.0842	0.043*	
C9	0.2815 (2)	0.1161 (4)	−0.10331 (11)	0.0375 (7)	
H9A	0.2625	0.1169	−0.1375	0.045*	
C10	0.3732 (2)	0.1366 (3)	−0.08306 (11)	0.0340 (6)	
H10A	0.4170	0.1505	−0.1035	0.041*	
C11	0.39861 (19)	0.1362 (3)	−0.03202 (10)	0.0285 (6)	
C12	0.49709 (19)	0.1659 (3)	−0.00939 (11)	0.0308 (6)	
C13	0.59738 (19)	0.1960 (3)	0.07196 (11)	0.0305 (6)	
C14	0.5979 (2)	0.1960 (3)	0.12313 (12)	0.0377 (7)	
H14A	0.5433	0.1866	0.1357	0.045*	
C15	0.6807 (2)	0.2147 (3)	0.05531 (12)	0.0360 (7)	
H15A	0.6831	0.2191	0.0215	0.043*	
C16	0.7602 (2)	0.2269 (4)	0.09021 (13)	0.0412 (7)	
H16A	0.8158	0.2380	0.0787	0.049*	
C17	0.6805 (2)	0.2102 (4)	0.15466 (12)	0.0424 (8)	
H17A	0.6798	0.2106	0.1887	0.051*	
Cd1	0.0000	0.06136 (3)	0.2500	0.03132 (12)	
H5A	0.133 (3)	0.229 (5)	0.3090 (16)	0.053 (12)*	
H6A	−0.088 (4)	−0.177 (5)	0.2195 (19)	0.068 (16)*	
H7A	0.441 (3)	1.003 (5)	0.1380 (18)	0.058 (13)*	
H2B	0.274 (3)	0.069 (4)	0.0709 (14)	0.039 (10)*	
H4B	0.470 (3)	0.153 (4)	0.0549 (15)	0.050 (11)*	
H5B	0.071 (3)	0.305 (4)	0.2858 (15)	0.044 (12)*	
H6B	−0.160 (4)	−0.097 (6)	0.218 (2)	0.09 (2)*	
H7B	0.415 (3)	0.948 (5)	0.0934 (19)	0.066 (16)*	
N1	0.07517 (18)	0.0563 (3)	0.18357 (10)	0.0358 (6)	
N2	0.21691 (18)	0.0606 (3)	0.06249 (9)	0.0306 (5)	
N3	0.33872 (16)	0.1131 (3)	−0.00068 (9)	0.0280 (5)	
N4	0.51311 (17)	0.1742 (3)	0.04068 (10)	0.0333 (6)	
N5	0.76221 (19)	0.2236 (3)	0.13927 (11)	0.0415 (6)	
O1	0.10075 (16)	0.0500 (3)	−0.00425 (9)	0.0533 (7)	
O2	0.55554 (15)	0.1802 (3)	−0.03618 (8)	0.0429 (6)	
O3	0.9969 (2)	0.4750 (3)	0.20625 (10)	0.0606 (8)	
O4	0.9169 (3)	0.6394 (5)	0.24461 (16)	0.1008 (14)	
O5	0.09316 (17)	0.2339 (3)	0.28544 (10)	0.0404 (5)	
O6	−0.10537 (19)	−0.1011 (3)	0.21310 (10)	0.0440 (6)	
O7	0.41102 (16)	1.0167 (3)	0.10871 (10)	0.0425 (6)	
O8	0.29730 (18)	0.8984 (3)	0.27805 (11)	0.0568 (7)	
H8B	0.3077	0.8417	0.2594	0.068*	
H8C	0.3420	0.9441	0.2797	0.068*	
S1	1.0000	0.56069 (11)	0.2500	0.0352 (2)	

### Tetraaquabis[N,N'-bis(pyridin-4-yl)pyridine-2,6-\ dicarboxamide]cadmium(II) sulfate tetrahydrate (II) Atomic displacement parameters (Å^2^)

**Table:**

	*U*^11^	*U*^22^	*U*^33^	*U*^12^	*U*^13^	*U*^23^
C1	0.0307 (15)	0.0508 (19)	0.0287 (15)	0.0013 (13)	0.0059 (12)	0.0002 (13)
C2	0.0257 (14)	0.053 (2)	0.0381 (17)	−0.0061 (13)	0.0093 (12)	−0.0052 (14)
C3	0.0271 (14)	0.0473 (19)	0.0322 (16)	−0.0053 (13)	0.0052 (12)	−0.0024 (13)
C4	0.0229 (13)	0.0478 (18)	0.0324 (15)	0.0003 (12)	0.0053 (11)	0.0005 (13)
C5	0.0283 (14)	0.0314 (15)	0.0306 (15)	0.0002 (11)	0.0096 (11)	−0.0007 (11)
C6	0.0272 (14)	0.0381 (16)	0.0308 (15)	−0.0031 (12)	0.0059 (11)	−0.0019 (12)
C7	0.0278 (14)	0.0311 (14)	0.0294 (14)	−0.0009 (11)	0.0065 (11)	−0.0013 (11)
C8	0.0295 (15)	0.0455 (18)	0.0300 (15)	−0.0024 (13)	−0.0002 (12)	−0.0002 (13)
C9	0.0408 (17)	0.0463 (18)	0.0237 (14)	−0.0014 (14)	0.0004 (12)	0.0002 (13)
C10	0.0355 (15)	0.0386 (16)	0.0298 (15)	−0.0006 (13)	0.0108 (12)	0.0005 (13)
C11	0.0267 (13)	0.0321 (15)	0.0274 (13)	0.0008 (11)	0.0070 (11)	0.0032 (11)
C12	0.0259 (13)	0.0360 (15)	0.0311 (14)	0.0010 (12)	0.0066 (11)	0.0010 (12)
C13	0.0259 (13)	0.0319 (15)	0.0332 (15)	−0.0029 (11)	0.0030 (11)	0.0016 (12)
C14	0.0323 (15)	0.0462 (19)	0.0353 (16)	−0.0061 (13)	0.0074 (12)	0.0005 (14)
C15	0.0309 (15)	0.0424 (17)	0.0351 (16)	−0.0044 (13)	0.0072 (12)	0.0025 (14)
C16	0.0288 (15)	0.0464 (19)	0.0484 (19)	−0.0065 (14)	0.0061 (13)	0.0000 (15)
C17	0.0440 (18)	0.049 (2)	0.0331 (16)	−0.0090 (15)	0.0018 (14)	−0.0002 (15)
Cd1	0.02672 (17)	0.0394 (2)	0.02952 (19)	0.000	0.00968 (12)	0.000
N1	0.0284 (13)	0.0469 (16)	0.0347 (14)	−0.0021 (11)	0.0129 (11)	−0.0017 (11)
N2	0.0226 (12)	0.0429 (15)	0.0273 (12)	−0.0019 (10)	0.0069 (10)	0.0009 (10)
N3	0.0258 (11)	0.0339 (13)	0.0252 (11)	0.0010 (10)	0.0071 (9)	0.0003 (10)
N4	0.0247 (12)	0.0453 (16)	0.0306 (13)	−0.0045 (11)	0.0071 (10)	0.0017 (11)
N5	0.0343 (14)	0.0443 (16)	0.0436 (15)	−0.0072 (12)	−0.0007 (12)	0.0000 (13)
O1	0.0270 (12)	0.096 (2)	0.0367 (13)	−0.0126 (12)	0.0036 (10)	−0.0016 (13)
O2	0.0326 (11)	0.0639 (17)	0.0348 (12)	−0.0061 (11)	0.0131 (9)	−0.0010 (11)
O3	0.096 (2)	0.0527 (17)	0.0318 (13)	−0.0022 (16)	0.0057 (14)	−0.0026 (12)
O4	0.093 (3)	0.111 (3)	0.104 (3)	0.059 (3)	0.032 (2)	0.014 (3)
O5	0.0357 (12)	0.0384 (13)	0.0426 (14)	0.0004 (11)	−0.0071 (10)	−0.0025 (11)
O6	0.0364 (13)	0.0438 (15)	0.0518 (15)	−0.0051 (11)	0.0077 (11)	−0.0037 (12)
O7	0.0332 (12)	0.0613 (17)	0.0326 (13)	0.0018 (11)	0.0038 (10)	0.0011 (12)
O8	0.0448 (14)	0.0694 (19)	0.0610 (17)	0.0013 (13)	0.0230 (13)	−0.0069 (14)
S1	0.0403 (6)	0.0326 (5)	0.0341 (6)	0.000	0.0102 (4)	0.000

### Tetraaquabis[N,N'-bis(pyridin-4-yl)pyridine-2,6-\ dicarboxamide]cadmium(II) sulfate tetrahydrate (II) Geometric parameters (Å, º)

**Table:**

C1—N1	1.338 (4)	C14—C17	1.375 (4)
C1—C4	1.373 (4)	C14—H14A	0.9300
C1—H1A	0.9300	C15—C16	1.386 (4)
C2—N1	1.344 (4)	C15—H15A	0.9300
C2—C3	1.377 (5)	C16—N5	1.335 (4)
C2—H2A	0.9300	C16—H16A	0.9300
C3—C5	1.397 (4)	C17—N5	1.344 (4)
C3—H3A	0.9300	C17—H17A	0.9300
C4—C5	1.395 (4)	Cd1—N1^i^	2.275 (3)
C4—H4A	0.9300	Cd1—N1	2.275 (3)
C5—N2	1.394 (4)	Cd1—O5^i^	2.334 (2)
C6—O1	1.218 (4)	Cd1—O5	2.334 (2)
C6—N2	1.359 (4)	Cd1—O6	2.371 (3)
C6—C7	1.503 (4)	Cd1—O6^i^	2.371 (3)
C7—N3	1.336 (4)	N2—H2B	0.84 (4)
C7—C8	1.397 (4)	N4—H4B	0.83 (4)
C8—C9	1.376 (5)	O3—S1	1.472 (3)
C8—H8A	0.9300	O4—S1	1.447 (3)
C9—C10	1.386 (4)	O5—H5A	0.79 (4)
C9—H9A	0.9300	O5—H5B	0.79 (4)
C10—C11	1.381 (4)	O6—H6A	0.82 (5)
C10—H10A	0.9300	O6—H6B	0.85 (6)
C11—N3	1.346 (4)	O7—H7A	0.86 (5)
C11—C12	1.508 (4)	O7—H7B	0.82 (5)
C12—O2	1.226 (4)	O8—H8B	0.8001
C12—N4	1.350 (4)	O8—H8C	0.8000
C13—C15	1.389 (4)	S1—O4^ii^	1.447 (3)
C13—C14	1.395 (4)	S1—O3^ii^	1.472 (3)
C13—N4	1.401 (4)		
			
N1—C1—C4	123.7 (3)	N5—C16—H16A	117.9
N1—C1—H1A	118.2	C15—C16—H16A	117.9
C4—C1—H1A	118.2	N5—C17—C14	123.9 (3)
N1—C2—C3	124.5 (3)	N5—C17—H17A	118.0
N1—C2—H2A	117.7	C14—C17—H17A	118.0
C3—C2—H2A	117.7	N1^i^—Cd1—N1	177.40 (15)
C2—C3—C5	118.1 (3)	N1^i^—Cd1—O5^i^	91.04 (10)
C2—C3—H3A	121.0	N1—Cd1—O5^i^	90.92 (10)
C5—C3—H3A	121.0	N1^i^—Cd1—O5	90.92 (10)
C1—C4—C5	119.2 (3)	N1—Cd1—O5	91.04 (10)
C1—C4—H4A	120.4	O5^i^—Cd1—O5	82.63 (14)
C5—C4—H4A	120.4	N1^i^—Cd1—O6	87.31 (10)
N2—C5—C4	117.6 (3)	N1—Cd1—O6	90.88 (10)
N2—C5—C3	124.3 (3)	O5^i^—Cd1—O6	92.82 (10)
C4—C5—C3	118.0 (3)	O5—Cd1—O6	175.09 (10)
O1—C6—N2	124.8 (3)	N1^i^—Cd1—O6^i^	90.88 (10)
O1—C6—C7	119.9 (3)	N1—Cd1—O6^i^	87.31 (10)
N2—C6—C7	115.3 (2)	O5^i^—Cd1—O6^i^	175.09 (10)
N3—C7—C8	122.8 (3)	O5—Cd1—O6^i^	92.82 (10)
N3—C7—C6	119.2 (3)	O6—Cd1—O6^i^	91.79 (15)
C8—C7—C6	118.0 (3)	C1—N1—C2	116.5 (3)
C9—C8—C7	118.9 (3)	C1—N1—Cd1	121.8 (2)
C9—C8—H8A	120.5	C2—N1—Cd1	121.7 (2)
C7—C8—H8A	120.5	C6—N2—C5	126.5 (3)
C8—C9—C10	118.6 (3)	C6—N2—H2B	118 (3)
C8—C9—H9A	120.7	C5—N2—H2B	116 (3)
C10—C9—H9A	120.7	C7—N3—C11	117.5 (2)
C11—C10—C9	119.0 (3)	C12—N4—C13	128.0 (3)
C11—C10—H10A	120.5	C12—N4—H4B	116 (3)
C9—C10—H10A	120.5	C13—N4—H4B	115 (3)
N3—C11—C10	123.1 (3)	C16—N5—C17	116.3 (3)
N3—C11—C12	117.4 (2)	Cd1—O5—H5A	126 (3)
C10—C11—C12	119.6 (3)	Cd1—O5—H5B	119 (3)
O2—C12—N4	125.0 (3)	H5A—O5—H5B	107 (4)
O2—C12—C11	120.0 (3)	Cd1—O6—H6A	114 (4)
N4—C12—C11	115.0 (2)	Cd1—O6—H6B	118 (4)
C15—C13—C14	117.9 (3)	H6A—O6—H6B	106 (5)
C15—C13—N4	124.2 (3)	H7A—O7—H7B	106 (5)
C14—C13—N4	117.9 (3)	H8B—O8—H8C	102.2
C17—C14—C13	119.0 (3)	O4^ii^—S1—O4	112.9 (4)
C17—C14—H14A	120.5	O4^ii^—S1—O3	108.8 (2)
C13—C14—H14A	120.5	O4—S1—O3	109.4 (2)
C16—C15—C13	118.5 (3)	O4^ii^—S1—O3^ii^	109.4 (2)
C16—C15—H15A	120.7	O4—S1—O3^ii^	108.8 (2)
C13—C15—H15A	120.7	O3—S1—O3^ii^	107.5 (2)
N5—C16—C15	124.3 (3)		
			
N1—C2—C3—C5	−1.2 (5)	C14—C13—C15—C16	2.7 (5)
N1—C1—C4—C5	0.6 (5)	N4—C13—C15—C16	−175.9 (3)
C1—C4—C5—N2	176.3 (3)	C13—C15—C16—N5	−1.0 (6)
C1—C4—C5—C3	−1.9 (5)	C13—C14—C17—N5	−0.4 (6)
C2—C3—C5—N2	−176.0 (3)	C4—C1—N1—C2	0.4 (5)
C2—C3—C5—C4	2.1 (5)	C4—C1—N1—Cd1	−179.3 (3)
O1—C6—C7—N3	176.6 (3)	C3—C2—N1—C1	−0.1 (5)
N2—C6—C7—N3	−2.2 (4)	C3—C2—N1—Cd1	179.6 (3)
O1—C6—C7—C8	−1.9 (5)	O1—C6—N2—C5	−7.0 (5)
N2—C6—C7—C8	179.3 (3)	C7—C6—N2—C5	171.6 (3)
N3—C7—C8—C9	2.8 (5)	C4—C5—N2—C6	−172.5 (3)
C6—C7—C8—C9	−178.7 (3)	C3—C5—N2—C6	5.6 (5)
C7—C8—C9—C10	−1.5 (5)	C8—C7—N3—C11	−1.8 (4)
C8—C9—C10—C11	−0.7 (5)	C6—C7—N3—C11	179.8 (3)
C9—C10—C11—N3	1.9 (5)	C10—C11—N3—C7	−0.6 (5)
C9—C10—C11—C12	−176.6 (3)	C12—C11—N3—C7	177.9 (3)
N3—C11—C12—O2	175.8 (3)	O2—C12—N4—C13	−2.2 (5)
C10—C11—C12—O2	−5.6 (5)	C11—C12—N4—C13	177.7 (3)
N3—C11—C12—N4	−4.0 (4)	C15—C13—N4—C12	0.9 (5)
C10—C11—C12—N4	174.6 (3)	C14—C13—N4—C12	−177.7 (3)
C15—C13—C14—C17	−2.1 (5)	C15—C16—N5—C17	−1.4 (6)
N4—C13—C14—C17	176.6 (3)	C14—C17—N5—C16	2.1 (6)

Symmetry codes: (i) −*x*, *y*, −*z*+1/2; (ii) −*x*+2, *y*, −*z*+1/2.

### Tetraaquabis[N,N'-bis(pyridin-4-yl)pyridine-2,6-\ dicarboxamide]cadmium(II) sulfate tetrahydrate (II) Hydrogen-bond geometry (Å, º)

**Table:**

*D*—H···*A*	*D*—H	H···*A*	*D*···*A*	*D*—H···*A*
N2—H2*B*···O7^iii^	0.84 (4)	2.17 (4)	2.958 (4)	156 (4)
N2—H2*B*···N3	0.84 (4)	2.35 (4)	2.736 (4)	109 (3)
N4—H4*B*···O7^iii^	0.83 (4)	2.29 (4)	3.030 (4)	150 (4)
N4—H4*B*···N3	0.83 (4)	2.29 (4)	2.698 (4)	111 (3)
O5—H5*A*···N5^iv^	0.80 (4)	1.91 (4)	2.704 (4)	174 (4)
O5—H5*B*···O3^iv^	0.79 (4)	2.02 (4)	2.811 (4)	172 (4)
O6—H6*A*···O4^v^	0.82 (5)	1.98 (5)	2.775 (6)	161 (6)
O6—H6*B*···O8^vi^	0.84 (6)	2.04 (6)	2.872 (4)	173 (6)
O7—H7*A*···O3^vii^	0.86 (5)	1.93 (5)	2.784 (4)	174 (4)
O7—H7*B*···O2^viii^	0.82 (5)	2.13 (5)	2.912 (4)	159 (5)
O8—H8*B*···O5^ix^	0.80	2.33	3.052 (4)	151
O8—H8*C*···O4^vii^	0.80	2.53	3.233 (6)	147
O8—H8*C*···O3^x^	0.80	2.36	3.085 (4)	152

Symmetry codes: (iii) *x*, *y*−1, *z*; (iv) −*x*+1, *y*, −*z*+1/2; (v) *x*−1, *y*−1, *z*; (vi) −*x*, *y*−1, −*z*+1/2; (vii) *x*−1/2, *y*+1/2, *z*; (viii) −*x*+1, −*y*+1, −*z*; (ix) −*x*+1/2, *y*+1/2, −*z*+1/2; (x) −*x*+3/2, *y*+1/2, −*z*+1/2.
